# A comparison of native and Syrian immigrant women students’ genital hygiene behaviors: a cross-sectional study

**DOI:** 10.1590/1980-220X-REEUSP-2025-0186en

**Published:** 2025-08-29

**Authors:** Sibel İçke, Sema Çifçi

**Affiliations:** 1 Mardin Artuklu University, Faculty of Health Sciences, Midwifery Department, Artuklu, Mardin, Türkiye.; 2 Mardin Artuklu University, Faculty of Health Sciences, Nursing Department, Artuklu, Mardin, Türkiye.

**Keywords:** Emigrants and immigrants, Genitalia, Health behavior, Hygiene, Students, Emigrantes e imigrantes, Genitália, Comportamentos Relacionados com a Saúde, Higiene; Estudantes

## Abstract

**Objective:**

This study aims to compare the genital hygiene behaviors of native and Syrian immigrant women students.

**Methods:**

This was cross-sectional research. The sample size of the study was determined via power analysis and G*Power software. A total sample size of 330 individuals was equally distributed to both groups. Sociodemographic Characteristics Questionnaire” and the ‘Genital Hygiene Behaviors Scale’ were used as data collection tools.

**Results:**

There was a significant difference between the mean scores of the scales of both student groups (p < 0.05). The mean scores of native students were found to be higher. The variables “how to clean the genital area”, “material used for genital hygiene”, “washing method of underwear”, “pretoilet hand washing habits” and “regular menstruation” were found to be most effective in terms of the mean scores of the Genital Hygiene Behaviors Scale and its subscales for immigrant students.

**Conclusion:**

Social security status and nationality were the most influential sociodemographic factors affecting genital hygiene behaviors. It would be beneficial to raise awareness and promote behavioral changes regarding genital hygiene through seminars, conferences, workshops, and similar events targeting all women university students.

## RESUMO

**Objetivo:**

Este estudo tem como objetivo comparar os comportamentos de higiene genital de estudantes mulheres nativas e imigrantes sírias.

**Método:**

Esta foi uma pesquisa transversal. O tamanho da amostra do estudo foi determinado por meio de análise de poder e do software G*Power. Uma amostra total de 330 indivíduos foi distribuída igualmente entre os dois grupos. O “Questionário de Características Sociodemográficas” e a “Escala de Comportamentos de Higiene Genital” foram usados como ferramentas de coleta de dados.

**Resultados:**

Houve uma diferença significativa entre as pontuações médias das escalas de ambos os grupos de estudantes (p < 0,05). As pontuações médias das estudantes nativas foram consideradas mais altas. As variáveis “como limpar a área genital”, “material usado para higiene genital”, “método de lavagem de roupas íntimas”, “hábitos de lavagem das mãos antes de usar o banheiro” e “menstruação regular” foram consideradas as mais eficazes em termos de pontuação média da Escala de Comportamentos de Higiene Genital e suas subescalas para estudantes imigrantes.

**Conclusão:**

O status de segurança social e a nacionalidade foram os fatores sociodemográficos mais influentes que afetaram os comportamentos de higiene genital. Seria benéfico aumentar a conscientização e promover mudanças comportamentais em relação à higiene genital por meio de seminários, conferências, workshops e eventos semelhantes voltados para todas as estudantes universitárias.

## INTRODUCTION

As a social phenomenon, migration is a complex issue. In general, migration refers to the movement of individuals from one place of residence to another for a variety of reasons. Factors such as war and climate change are among the main drivers of migration^(1)^. Since 2011, as a result of the ongoing civil war and conflict in Syria, hundreds of thousands of citizens of Syria have been forced to migrate to Turkey. According to the Directorate General of Migration Management under the Ministry of the Interior of the Republic of Turkey, as of June 2025, the number of Syrians under temporary protection in Turkey was 2,699,787, with 45,273 residing in the province of Mardin^(2)^.

Migration affects migrants in many areas, one of which is health. Migrants face different health needs depending on the living conditions they have in their own countries and the conditions they encounter in the countries to which they migrate^(1)^. In their new environments, they frequently encounter barriers in accessing healthcare services. Language difficulties, a lack of health insurance, and insufficient knowledge about healthcare systems are among the major obstacles to obtaining sustainable health services^(3)^.

Recently, migration has increasingly been examined in relation to gender, especially in studies focusing on women. Given the negative impacts of migration on women’s health, women can be considered particularly vulnerable^(1,4)^. Migrant women are at risk in terms of their sexual and reproductive health needs. Gender, as a sociocultural determinant, is a key factor in this risk. Gender inequality, one of the social determinants of health, affects health status and needs, ways of accessing healthcare services, and the provision of healthcare services in various ways. Gender inequality is particularly evident in the field of health and is most noticeable in reproductive health services^(5)^.

Early marriage, adolescent and older pregnancies, excessive fertility, lack of access to family planning services and infections due to a lack of genital hygiene are among the most common problems faced by migrant women^(6)^. A study evaluating reproductive health issues among Syrian refugee women revealed that 60% of women had pathological discharge complaints^(7)^. According to the 2019 Migration and Health Report of the Ministry of Health of the Republic of Turkey, there is an increased risk of genital and urinary tract infections among refugees and asylum seekers^(8)^. The UNFPA Turkiye performs activities aimed at empowering women and girls and distributing hygiene kits and maternal and reproductive health kits to refugees^(9)^. This study was developed on the basis of the assumption that evaluating the effectiveness of hygiene-related interventions (e.g., education, hygiene kits) for migrants is essential for emphasizing the sustainability of aid efforts. There are studies in Turkey and around the world on determining women’s genital hygiene behaviors, researching the factors that influence genital hygiene behaviors, and evaluating the relationship between genital hygiene behaviors and genital infections^(10,11,12,13,14,15,16,17,18)^. Although university students do not represent all migrant women, this group is considered to be more advantaged in terms of access to healthcare services and education level. Therefore, the findings are expected to provide insights into a specific sociocultural segment. The aim of this study was to compare the genital hygiene behaviors of native and Syrian migrant female students at Mardin Artuklu University and to identify the cultural and sociodemographic factors (such as age, income level, and marital status) that influence these behaviors.

## METHOD

### Type of Research

This study used a cross-sectional research design to compare the genital hygiene behaviors of native and Syrian migrant women students studying at Mardin Artuklu University and to determine the cultural and sociodemographic factors (age, income status, marital status, etc.) that influence these behaviors. The cross-sectional design was selected because it enables the practical assessment of health behaviors across a broader sample, particularly when longitudinal follow-up studies are limited by challenges such as access, mobility, time constraints, and difficulties in obtaining informed consent—common issues when researching migrant populations. In public health research, cross-sectional studies are frequently preferred to examine associations between demographic variables and health behaviors. They are particularly valuable for identifying current conditions and the role of social determinants in shaping health- related outcomes.

### Participants and Sampling

The study population consisted of women students (n = 5970) enrolled in nonhealth departments (since it was assumed that health department students had received training on genital hygiene) at Mardin Artuklu University from 2022-2023. Of these, 636 were Syrian women students. The sample size of the study was determined via power analysis and G*Power software. A total sample size of 330 individuals was equally distributed to both groups, with a significance level of p = 0.05, a medium effect size, a study power of 99%, and a 1-Beta value set to 0.99 on the basis of Cohen’s d = 0.5^(19)^. Considering time and resource constraints, the convenience sampling method was chosen to reach the target audience quickly and efficiently. While this choice ensured the practicality of the study, it also limited the generalizability of the findings, as the data were focused on a specific group of students at Mardin Artuklu University.

### Ethical Consideration

All necessary permission was obtained from the Non-Invasive Ethics Committee of the Rectorate of Mardin Artuklu University (date: November 9, 2022, no: 2022/13-24) and from Karahan, who developed the Genital Hygiene Behaviors Scale (GHBS) and assessed its validity and reliability. The study was conducted in compliance with the principles outlined in the Declaration of Helsinkİ Prior to data collection, the participants were provided with the necessary information about the research, and written informed consent was obtained.

### Data Collection Tools

The data collection tool consisted of two sections. In the first section, the Sociodemographic Characteristics Questionnaire (SCQ) was used to determine the sociodemographic characteristics of the participants included in the study, whereas in the second section, the GHBS was used to assess genital hygiene behaviors.

– SCQ: The questionnaire was developed by the researchers only for this study. The SCQ consists of a total of 35 questions related to the student’s age, nationality, place of residence, citizenship status if foreign, marital status, whether they have children, family type, social security status, parents’ educational level, the student’s employment status, income status, the region where they reside during their education, smoking habits, presence of chronic diseases, and genital hygiene behaviors^(13,14,17,20,21)^.
– GHBS: This Likert-type scale was developed by Karahan in 2017, and its reliability and validity were assessed. The scale has been used previously in different populations and is a widely used tool to assess women’s genital hygiene behaviors. It consists of 23 statements rated on a scale ranging from “Strongly agree” to “Strongly disagree,” ranging from 5-1. The scale comprises three subscales: “General Hygiene Habits (GHH)” (first 12 statements), “Menstrual Hygiene Habits (MHH)” (13-20 statements), and “Awareness of Abnormal Findings (AAF)” (21–23 statements). In the evaluation, statements 7, 14, 19, 20, and 23 are reverse-coded, allowing for a score range of 23 (lowest) to 115 (highest), with higher scores indicating better genital hygiene behaviors in women. The Cronbach’s alpha value of the original scale was 0.80^(20)^. In our study, the Cronbach’s alpha value for the scale was 0.83.

### Data Collection Method

The study data were collected between March and May 2023 via face‒to-face interviews. Participants who met the inclusion criteria were informed about the study, and both written and verbal consent were obtained. The data collection forms took approximately 10–15 minutes to complete.

### Inclusion Criteria

– Woman students aged 18 and above
– Accepting to participate in the research voluntarily
– Studying in departments other than health departments
– Not having Turkish language problems (for Syrian students)

### Data Analysis

After the researcher coded the data from this study, they were transferred to SPSS version 16.0 software to conduct the necessary analyses.

The statistical analysis of the study involved the use of means, standard deviations, minimum and maximum values, and percentile numbers. To determine whether the data were normally distributed, the Kolmogorov‒Smirnov test was used. Normally distributed scores were evaluated via chi-square tests, t tests (independent samples t tests) and one-way ANOVA. Tukey’s test, a post hoc test, was applied to determine which groups were different from each other via one-way ANOVA. The data were analyzed via a general linear model (GLM) to control for potential confounding variables.

## RESULTS

The mean age of the students who participated in the study was 23.02 ± 4.42 years (Min: 17, Max: 50). The mean age of the native students was 23.12 ± 4.27 years (Min: 17, Max: 48), while the mean age of the immigrant students was 23.06 ± 4.72 years (Min: 18, Max: 50).

The sociodemographic characteristics of the participants are presented in Table 1.

**Table 1:** Sociodemographic characteristics of the participants – Mardin, Türkiye, 2023.

Characteristics		Native students		Immigrant students		X^2^	p
		n	%	n	%		
Marital status	Married	16	9.7	32	19.4	6.24	**< 0.05**
	Single	149	90.3	133	80.6		
Having children	Yes	11	6.7	26	15.8	6.84	**< 0.05**
	No	154	93.3	139	84.2		
Mother’s education level	Illiterate	42	25.5	29	17.6	15.33	**< 0.05**
	Primary school graduate	58	35.2	36	21.8		
	Secondary school graduate	25	15.2	37	22.4		
	High school graduate	24	14.5	34	20.6		
	Bachelor’s degree and above	16	9.7	29	17.6		
Father’s education level	Illiterate	7	4.2	12	7.3	19.79	**< 0.05**
	Primary school graduate	47	28.5	18	10.9		
	Secondary school graduate	29	17.6	48	29.1		
	High school graduate	43	26.1	40	24.2		
	Bachelor’s degree and above	39	23.6	47	28.5		
Area of residence during education	Public dormitory	89	53.9	48	29.1	29.97	**< 0.05**
	Private dormitory	7	4.2	20	12.1		
	Student house	15	9.1	40	24.2		
	Together with family	54	32.7	57	34.5		
Social security	Has social security	105	63.6	59	35.8	25.64	**< 0.05**
	Hasn’t social security	60	36.4	106	64.2		
Total		165	100.0	165	100.0		

X^2^: chi-square test.

There are statistically significant differences between native and immigrant students in terms of marital status, having children, parents’ educational levels, the area of residence during education, and social security coverage characteristics (p < 0.05).

Compared with native students, migrant students are more likely to be married and have children (19.4%). Additionally, the educational levels of migrant students’ parents are generally higher than those of native students’ parents. During the academic year, the percentage of migrant students residing in public dormitories (29.1%) was lower than that of native students, whereas the percentages residing in private dormitories (12.1%) and student homes (24.2%) is higher. The percentage of migrant students without social security coverage is higher than that of native students (64.2%).

The students’ behaviors related to genital hygiene are presented in Table 2.

**Table 2:** Students’ (N = 330) behaviors related to genital hygiene – Mardin, Türkiye, 2023.

Characteristics		Native students		Immigrant students		X^2^	p
		n	%	n	%		
Receiving genital hygiene education	Yes	59	35.8	82	49.7	6.80	**< 0.05**
	No	77	46.7	63	38.2		
	Yes, but insufficient	29	17.6	20	12.1		
	Randomly	20	12.1	30	18.2		
Having genital or urinary tract infection	Yes	67	40.6	45	27.3	6.54	**< 0.05**
	No	98	59.4	120	72.7		
Regular menstruation	Yes	144	87.3	127	77.0	5.96	**< 0.05**
	No	21	12.7	38	23.0		
Use of any substance, cream, perfume, etc., on the genital area	Yes	12	7.3	26	15.8	5.82	**< 0.05**
	No	153	92.7	139	84.2		
The method of genital hair removal	Wax	31	18.8	46	27.9	13.23	**< 0.05**
	Razor blade	69	41.8	79	47.9		
	Epilator	46	27.3	27	16.4		
	Clipper	2	1.2	5	3.0		
	No hair	18	10.9	8	4.8		
Presence of foul-smelling discharge	Yes	41	24.8	16	9.7	13.25	**< 0.05**
	No	124	75.2	149	90.3		
Training on abnormal discharge	Yes	21	12.7	50	30.3	15.9	**< 0.05**
	No	144	87.3	115	69.7		
Washing method of underwear	At high temperature in the machine	127	77.0	132	80.0	6.45	**< 0.05**
	At low temperature in the machine	31	18.8	18	10.9		
	By hand/by boiling	7	4.2	15	9.1		
Total		165	100.0	165	100.0		

X^2^: chi-square test.

Significant differences in hygiene and health habits were detected between native and migrant students. Migrant students had higher rates of genital hygiene education (49.7%). The rates of genital or urinary tract infections (40.6%) and regular menstruation (87.3%) were higher among native students. The rates of using any substance in the genital area (15.8%) and using a razor to remove hair in the genital area (47.9%) were higher among migrant students. The rate of foul smelling discharge was higher in native students (24.8%), and the rate of receiving education about abnormal discharge was higher in migrant students (30.3%). With respect to the method of washing underwear, migrant students use machine washing at high temperature (80.0%).

The descriptive statistical distributions of the scores of the GHBS and its subscales are presented in Table 3.

**Table 3:** Descriptive statistical distribution of the scores of the GHBS and its subscales – Mardin, Türkiye, 2023.

Scale and subscales	Native Students					Immigrant Students					*p Value
	N	Min.	Max.	Mean	Sd	N	Min.	Max.	Mean	Sd	
GHBS	165	51.00	114.00	90.81	11.96	165	44.00	113.00	86.50	14.01	**< 0.05**
GHH	165	24.00	60.00	46.63	6.29	165	17.00	60.00	45.24	8.21	> 0.05
MHH	165	17.00	40.00	33.48	4.89	165	14.00	40.00	30.07	5.28	**< 0.05**
AAF	165	3.00	15.00	10.78	3.45	165	5.00	15.00	11.18	2.53	> 0.05

GHH: general hygiene habit, MHH: menstrual hygiene habit, AAF: awareness of abnormal findings.

*Independent samples t test.

While there was a statistically significant difference between the two groups in the GHBS and MHH subscale scores (p < 0.05), there was no statistically significant difference between the two groups in the GHH and AAF subscale scores (p > 0.05).

The data were analyzed via a general linear model (GLM) to control for potential confounding variables and are presented in Table 4.

**Table 4:** The effects of sociodemographic characteristics as confounding factors on genital hygiene behaviors – Mardin, Türkiye, 2023.

Source	Sum of Squares	df	Mean Square	F	Sig.	Partial Eta Squared
Corrected Model	4439.553	7	634.222	3.903	**< 0.05**	0.078
Intercept	452629.631	1	452629.631	280.807	**< 0.05**	0.466
Marital Status	8.406	1	8.406	0.052	> 0.05	0.000
Do you have children?	9.253	1	9.253	0.057	> 0.05	0.000
Mother’s Education Level	383.658	1	383.658	2.361	> 0.05	0.007
Father’s Education Level	98.255	1	98.255	0.605	> 0.05	0.002
Where do you stay during the education period?	86.189	1	86.189	0.531	> 0.05	0.002
Do you have social security?	1414.934	1	1414.934	8.708	**< 0.05**	0.026
Nationality	1144.861	1	1144.861	7.046	**< 0.05**	0.021
Error	52233.344	322	162.495			
Total	56762.897	329				

Social security and nationality were found to have a significant confounding effect on genital hygiene behaviors (p < 0.05). Students with social security and native-born students had significantly higher genital hygiene behavior scores. The partial eta square (η^2^) shows that the confounding effects of the social security (2.6%) and nationality (2.1%) variables are small (η^2^ < 0.06). Other variables (marital status, having children, parental education level and place of residence during the education period) did not significantly confound genital hygiene behaviors (p > 0.05).

Table 5 presents the comparison of total scale and subscale scores between native and immigrant students on the basis of certain characteristics.

**Table 5:** Comparison of total scale and subscale scores between native and immigrant students on the basis of certain characteristics – Mardin, Türkiye, 2023.

Characteristics		Native Students				Immigrant Students			
		GHBS	GHH	MHH	AAF	GHBS	GHH	MHH	AAF
Age*	17–21 Age^a^	89.23 ± 12.99	45.25 ± 6.76	33.50 ± 5.24	10.47 ± 3.63	86.09 ± 13.40	45.02 ± 8.04	30.36 ± 5.04	10.70 ± 2.49
	22–26 Age^b^	90.45 ± 11.16	46.80 ± 5.93	33.21 ± 4.85	10.43 ± 3.38	85.55 ± 13.65	44.86 ± 7.98	29.48 ± 5.34	11.20 ± 2.30
	27–31 Age^c^	102.40 ± 7.64	52.30 ± 5.16	36.70 ± 3.43	13.40 ± 2.11	92.45 ± 13.95	48.09 ± 9.19	31.90 ± 3.59	12.45 ± 2.94
	32 Age^d^ ↑	92.25 ± 9.32	46.91 ± 5.23	32.66 ± 3.84	12.66 ± 2.60	88.91 ± 19.36	46.16 ± 10.0	29.91 ± 7.51	12.83 ± 2.82
	Statistical Analysis	F = 3.79 **p = 0.01**	F = 3.79 **p = 0.01**	F = 1.66 p = 0.17	F = 3.73 **p = 0.01**	F = 0.90 p = 0.40	F = 0.55 p = 0.64	F = 0.790 p = 0.49	F = 3.67 **p = 0.01**
	Difference	a–c, b–c, c–a–b	a–b–c, b–a–c, c–a–b		b–c, c–b				a–d
Income status*	Income less than expenditure^a^	87.17 ± 12.31	45.40 ± 6.36	32.56 ± 4.78	9.21 ± 3.56	85.43 ± 14.61	44.45 ± 8.73	29.76 ± 5.33	11.08 ± 2.66
	Income equals expenditure^b^	91.90 ± 11.88	46.94 ± 6.37	33.64 ± 5.00	11.32 ± 3.08	87.81 ± 13.50	46.20 ± 7.74	30.38 ± 5.13	11.22 ± 2.43
	Income more than expenditure^c^	96.25 ± 10.39	48.45 ± 5.46	35.12 ± 4.41	12.66 ± 2.94	83.46 ± 14.28	42.61 ± 8.17	29.46 ± 6.29	11.38 ± 2.69
	Statistical Analysis	F = 5.91 p = **0.003**	F = 2.22 p = 0.111	F = 2.45 p = 0.089	F = 11.91 p = **0.000**	F = 0.87 p = 0.421	F = 1.48 p = 0.229	F = 0.33 p = 0.719	F = 0.09 p = 0.908
	Difference	a–b–c, b–a, c–a			a–b–c, b–a, c–a				
Marital status**	Married	93.50 ± 11.87	48.18 ± 6.02	32.62 ± 5.76	12.68 ± 2.57	87.59 ± 16.60	45.28 ± 9.86	30.09 ± 5.58	12.21 ± 2.81
	Single	90.62 ± 11.83	46.46 ± 6.32	33.57 ± 4.80	10.58 ± 3.47	86.24 ± 13.37	45.24 ± 7.81	30.06 ± 5.23	10.93 ± 2.40
	Statistical Analysis	t = 0.92 p = 0.357	t = 1.04 p = 0.299	t = 0.74 p = 0.461	t = 2.35 **p = 0.020**	t = 0.49 p = 0.625	t = 0.25 p = 0.980	t = 0.25 p = 0.980	t = 2.38 **p = 0.022**
Family type**	Extended	87.76 ± 11.42	45.61 ± 6.16	32.46 ± 4.51	9.68 ± 3.84	83.94 ± 15.76	43.40 ± 9.50	29.69 ± 5.92	10.84 ± 2.67
	Nuclear (mother, father and children)	92.15 ± 11.80	47.03 ± 6.33	33.88 ± 4.99	11.22 ± 3.19	87.92 ± 12.80	46.27 ± 7.25	30.28 ± 4.91	11.36 ± 2.45
	Statistical Analysis	t = –2.17 **p = 0.031**	t = –1.31 p = 0.193	t = –1.69 p = 0.092	t = –2.65 **p = 0.009**	t = 1.66 p = 0.101	t = –2.01 **p = 0.047**	t = –0.65 p = 0.518	t = –1.27 p = 0.207
Social security**	Has social security	92.58 ± 10.63	47.48 ± 5.71	33.92 ± 4.19	11.17 ± 3.49	89.84 ± 12.17	47.00 ± 7.11	31.11 ± 4.95	11.72 ± 2.63
	Hasn’t social security	87.96 ± 13.25	45.13 ± 7.00	32.71 ± 5.88	10.11 ± 3.29	84.64 ± 14.66	44.27 ± 8.64	29.49 ± 5.40	10.87 ± 2.43
	Statistical Analysis	t = 2.30 **p = 0.023**	t = 2.33 **p = 0.021**	t = 1.40 p = 0.165	t = 1.90 p = 0.059	t = 2.44 **p = 0.016**	t = 2.18 **p = 0.031**	t = 1.91 p = 0.058	t = 2.09 **p = 0.038**
Use of any substance, cream, perfume, etc., on the genital area	Yes	96.66 ± 8.53	50.58 ± 3.60	34.08 ± 5.20	12.00 ± 2.95	82.04 ± 19.03	42.00 ± 11.63	29.35 ± 6.55	10.69 ± 2.91
	No	90.45 ± 11.95	46.32 ± 6.37	33.44 ± 4.88	10.69 ± 3.48	87.34 ± 12.78	45.86 ± 7.30	30.21 ± 5.04	11.27 ± 2.46
	Statistical Analysis	t = 1.76 p = 0.08	t = 3.67 **p = 0.00**	t = 0.44 p = 0.66	t = 1.27 p = 0.21	t = –1.36 p = 0.18	t = –1.63 p = 0.11	t = –0.64 p = 0.53	t = –1.07 p = 0.29
The method of genital hair removal*	Wax^a^	89.77 ± 14.60	45.54 ± 7.29	33.38 ± 5.10	10.83 ± 3.64	88.95 ± 15.05	46.50 ± 8.44	30.73 ± 5.68	11.71 ± 2.44
	Razor blade ^b^	87.95 ± 11.37	45.26 ± 6.23	32.50 ± 4.83	10.18 ± 3.45	85.67 ± 13.20	44.94 ± 7.77	29.92 ± 4.96	10.79 ± 2.52
	Epilator^c^	94.04 ± 9.09	48.28 ± 5.70	34.51 ± 4.09	11.24 ± 3.41	87.81 ± 12.29	46.37 ± 7.63	30.11 ± 4.89	11.33 ± 2.61
	Clipper ^d^	82.50 ± 20.50	43.00 ± 2.82	28.50 ± 12.08	11.00 ± 5.65	69.40 ± 13.52	35.40 ± 10.73	24.20 ± 2.94	9.80 ± 2.48
	No hair	97.22 ± 10.17	50.00 ± 4.22	35.38 ± 5.08	11.83 ± 2.93	86.87 ± 16.50	43.37 ± 8.81	31.25 ± 7.06	12.25 ± 2.37
	Statistical Analysis	F = 3.70 **p = 0.007**	F = 3.48 **p = 0.009**	F = 2.47 **p = 0.046**	F = 1.14 p = 0.341	F = 2.42 **p = 0.050**	F = 2.40 **p = 0.050**	F = 1.88 p = 0.117	F = 1.75 p = 0.142
	Difference	b–e	b–e	e–b		a–d	a–d, c–d		
Washing method of underwear *	At high temperature in the machine ^a^	91.51 ± 11.14	46.93 ± 6.05	33.54 ± 4.77	11.03 ± 3.26	88.13 ± 13.84	46.03 ± 8.24	30.68 ± 5.02	11.41 ± 2.44
	At low temperature in the machine^b^	86.87 ± 13.12	44.51 ± 6.66	32.61 ± 5.27	9.74 ± 4.00	78.11 ± 11.44	41.72 ± 6.71	26.77 ± 5.66	9.61 ± 2.72
	By hand/by boiling^c^	97.71 ± 11.25	50.42 ± 7.13	36.28 ± 4.71	11.00 ± 3.26	82.20 ± 14.59	42.60 ± 8.39	28.60 ± 5.69	11.00 ± 2.56
	Statistical Analysis	F = 3.21 **p = 0.043**	F = 3.26 **p = 0.041**	F = 1.66 p = 0.193	F = 1.77 p = 0.174	F = 5.07 **p = 0.007**	F = 3.11 **p = 0.047**	F = 5.23 **p = 0.006**	F = 4.22 p = 0.016
	Difference	a–b	b–a			a–b	a–b	a–b	
Regular menstruation**	Yes	91.40 ± 11.77	46.85 ± 6.29	33.72 ± 4.77	10.81 ± 3.42	88.53 ± 13.22	46.41 ± 7.80	30.77 ± 5.02	11.33 ± 2.50
	No	87.47 ± 11.91	45.09 ± 6.24	31.80 ± 5.44	10.57 ± 3.69	79.71 ± 14.61	41.34 ± 8.44	27.71 ± 5.54	10.65 ± 2.60
	Statistical Analysis	t = 1.425 p = 0.156	t = 1.197 p = 0.233	t = 1.690 p = 0.093	t = 0.307 p = 0.759	t = 3.522 **p = 0.001**	t = 3.450 **p = 0.001**	t = 3.226 **p = 0.002**	t = 1.457 p = 0.680
Presence of foul–smelling discharge**	Yes	85.80 ± 11.57	45.90 ± 6.64	31.87 ± 4.73	8.02 ± 3.51	78.93 ± 13.01	41.93 ± 7.66	27.31 ± 5.91	9.68 ± 2.98
	No	92.58 ± 11.46	46.87 ± 6.19	34.01 ± 4.84	11.70 ± 2.91	87.31 ± 13.91	45.60 ± 8.21	30.36 ± 5.15	11.34 ± 2.44
	Statistical Analysis	t = 3.28 **p = 0.001**	t = –0.85 p = 0.395	t = 2.46 **p = 0.015**	t = 6.65 **p = 0.000**	t = 2.30 **p = 0.023**	t = 1.71 p = 0.090	t = 2.22 **p = 0.028**	t = 2.52 **p = 0.013**

*One-way ANOVA and Tukey’s test (post hoc).

**Independent samples t test.

In the present study, genital hygiene behaviors and their subscales were not influenced by variables such as father’s education level, mother’s education level, having children, area of residence during education, employment status, receiving genital hygiene education, type of toilet used, pain during menstruation, use of any substance, cream, perfume, etc., in the genital area, or training on abnormal discharge (p > 0.05).

## DISCUSSION

In our findings, the most statistically significant differences in genital hygiene behaviors and subscales in both native and immigrant individuals were found in terms of the method of cleaning the genital area, the washing method of underwear, social security, and the presence of foul-smelling discharge. The fact that aid work and projects have been carried out for migrants in Turkey may have a positive effect on some outcomes^(22)^.

There was a difference between the income status of native students and the mean scores of the GHBS and the awareness of abnormal findings subscale scores. Studies conducted in different locations^(14,21)^ have shown that as income increases, the total scores of the genital hygiene behavior scale also increase. The fact that it is easier for those with higher incomes to access hygienic products may have positively affected their genital hygiene behaviors.

While there were significant differences between native students’ family type, general hygiene habits, and awareness of abnormal findings subscale scores, such differences were observed in immigrants’ general hygiene habits subscale scores. The total mean scores of those living in nuclear families were higher. The nuclear family, defined as a family unit consisting solely of parents and their children, may have a positive impact on hygiene-related behaviors. This structure facilitates greater privacy for individuals, enhances access to hygiene products, and reduces the number of people sharing communal spaces such as bathrooms and toilets, thereby promoting improved hygiene practices. In a study conducted with adolescent girls attending school in Nepal, 67% of the participants had a nuclear family. Among those with one to four family members, the percentage of individuals exhibiting strong menstrual hygiene practices was greater than that among those with five or more family members^(19)^. In a study examining the genital hygiene behaviors of women visiting the Community Health Center in Mardin, women with nuclear families had higher mean genital hygiene behavior scores^(18)^. The results of our study mostly align with the findings in the literature.

In our study, the mean scores of the genital hygiene behaviors scale and its subscales were generally higher in those with social security. In different studies conducted on disadvantaged and nondisadvantaged women, the scores of genital hygiene behaviors of those with social security were found to be higher, as in our study^(12,18,22)^. The lower cost of health care for those with social security directs them to health institutions and allows them to access the necessary information from health professionals.

In the present study, a total of 41.8% of the native students and 47.9% of the migrant students reported that they cleaned their genital areas with razor blades. In a study in which women students living in a state dormitory participated, the percentage of those who cleaned their genital areas with razor blades (35.9%) was greater than that of those who cleaned their genital areas with wax (34.3%)^(21)^. Factors such as the economic opportunities of the students, the necessity of living together in crowded places and a lack of knowledge about genital hygiene may have affected the results obtained. This study did not evaluate a direct relationship between hair removal methods and infection rates; however, the literature reports that razor use may increase the risk of infection by damaging skin integrity (itching, irritation, localized eczema, ingrown hairs, microscopic cuts, etc.)^(23,24)^. Therefore, the high rate of razor use is noteworthy in terms of potential risk. Since the study has a cross-sectional design, establishing a causal relationship is not possible; however, this situation can be considered an important topic for future research.

Native students who handwash their underwear with boiling water and immigrant students who wash it at high temperatures on machines generally have higher scores on the GHBS and its subscales. There may be two possible explanations for this finding. First, migrants who have not acquired Turkish citizenship are not eligible to reside in public dormitories and therefore may be living in private dormitories, student housing, or together with families—settings that may offer better hygiene conditions than public dormitories. Second, their responses may reflect accurate knowledge and improved hygiene practices gained through educational components of health-oriented humanitarian aid programs. In a study by Baş et al.^(25)^, which was conducted at credit and dormitory institutions, 54.80% of the students were, and in a study by Kartal et al.^(17)^, which was conducted on midwifery students, 91.40% of the students stated that they washed their underwear in the washing machine. In a study by Toraman et al.^(26)^ examining the genital hygiene behaviors of women living in a women guest house, 20.70% of women washed their underwear at high temperatures in the machine. The studies in the literature may show variabilities, which may be attributed to limited resources, cultural differences, and insufficient knowledge on the subject.

The rate of menstrual irregularities among immigrant students is 23%, whereas it is 12.7% among native students. Those who experienced regular menstruation had higher GHBS, general hygiene habits, and total menstrual hygiene scores. Menstrual irregularities in immigrant students may be related to psychological stress, barriers to accessing health services, and cultural adaptation issues, primarily economic difficulties. In a study by Janoowalla et al.^(27)^ conducted in Rwanda, 69.10% of adolescents, and in a study by Belayneh and Mekuriaw^(28)^ examining the menstrual hygiene knowledge of adolescents in southern Ethiopia, 40.30% reported experiencing regular menstruation. The fact that both countries in the sample studies have very low socioeconomic levels suggests that menstrual irregularities may be caused by problems resulting from economic difficulties in particular. Our study is consistent with the literature. It would be beneficial to establish comprehensive support mechanisms through collaboration between universities, nongovernmental organizations (NGOs), and the Ministry of Health to solve these problems.

Among all the students, 75.20% of the native students and 90.30% of the immigrant students reported that they did not have foul-smelling discharge. The presence of foul-smelling discharge generally affects genital hygiene behaviors, menstrual hygiene, and awareness of abnormal findings. Muzayyanatul and Wulan^(29)^ reported a lower rate of pathological vaginal discharge (3.50%) among students who maintained genital hygiene. In a study on the prevention of genitourinary tract infections among female adolescent students, vaginal discharge was reported to account for 18.60% of the reported complaints^(30)^. Interestingly, some hygiene behaviors among Syrian refugee students have been observed to be more positive than expected. Considering the socioeconomic challenges faced by migrant groups, this situation may seem contradictory at first glance. However, health- focused aid projects, awareness seminars, and informational activities targeting Syrian refugees living in Turkey may have supported the cultural adaptation process by encouraging the adoption of local health habits. On the other hand, since the data used in our study are self-reported, the possibility that participants may be inclined to give socially acceptable responses, especially on sensitive issues (reporting bias), should also be considered.

### Study Limitations

- This study is limited to a sample of university students. The education levels of native and migrant women may differ, which may affect their hygiene habits.
- Since the data are based on students’ self-reports, they may have given answers that were considered more socially acceptable. This may lead to bias.
- In this study, the length of stay in Turkey and the level of cultural adaptation of immigrant students were not included in the scope of the evaluation. However, these variables have the potential to affect both hygiene behaviors and participants’ self-reports. Taking these factors into account in future studies will enable a more comprehensive and in-depth analysis of the findings.

## CONCLUSION

In conclusion, the differences in hygiene habits between native and immigrant students may be due to cultural, educational, socioeconomic, etc., factors. The development and sustainability of education and health policies that take these differences into account can play an important role in improving the hygiene standards of native and migrant students.

Studies comparing the hygiene habits of native and immigrant students remain limited. Additionally, because this study utilized a cross-sectional design, it could not capture behavioral changes over time. Therefore, future longitudinal studies evaluating the effectiveness of hygiene education and targeted interventions are recommended to better understand temporal trends and support evidence-based health practices among diverse student populations.

Intervention-based educational programmes to be developed in the future for both native and migrant students offer an important area of research in terms of strengthening genital hygiene behaviors and creating sustainable awareness. In this context, developing health promotion strategies targeting migrant populations is highly important. Culturally sensitive educational programs, public policies that ensure access to hygiene products and services—especially for women without social security—and awareness campaigns aimed at reducing stigma can improve hygiene conditions and help reduce inequalities in the field of reproductive health among these vulnerable groups.

## Data Availability

The data that support the findings of this cross-sectional study were collected through face-to-face interviews using a Sociodemographic Characteristics Questionnaire and the Genital Hygiene Behaviors Scale (GHBS). These datasets are not publicly available due to participant confidentiality and ethical restrictions, but they are available from the corresponding author upon reasonable request.
