# Supplementary material for: A comparison of native and Syrian immigrant women students’ genital hygiene behaviors: a cross-sectional study
Source: Rev Esc Enferm USP. 2025 Aug 29;59:e20250186. doi: 10.1590/1980-220X-REEUSP-2025-0186en (PMC12395602; doi:10.1590/1980-220X-REEUSP-2025-0186en)
Supplement: Translation

# Translation: Comparación entre los hábitos de higiene genital de las estudiantes nativas turcas y las inmigrantes sirias: un estudio transversal

**DOI:** 10.1590/1980-220X-REEUSP-2025-0186es

**Authors:** Sibel İçke, Sema Çifçi

**Affiliations:** 3 Mardin Artuklu University, Faculty of Health Sciences, Midwifery Department, Artuklu, Mardin, Türkiye.; 4 Mardin Artuklu University, Faculty of Health Sciences, Nursing Department, Artuklu, Mardin, Türkiye.

**Keywords:** Emigrantes e inmigrantes, Genitales, Conductas Relacionadas con la Salud, Higiene, Estudiantes

## Abstract

**Objetivo:**

El objetivo de este estudio es comparar los hábitos de higiene genital de las estudiantes nativas y las inmigrantes sirias.

**Método:**

Se trata de una investigación transversal. El tamaño de la muestra del estudio se determinó mediante un análisis de potencia y el software G*Power. El tamaño total de la muestra fue de 330 personas, distribuidas equitativamente entre ambos grupos. Como herramientas de recopilación de datos se utilizaron el “Cuestionario de características sociodemográficas” y la “Escala de hábitos de higiene genital”.

**Resultados:**

Se observó una diferencia significativa entre las puntuaciones medias de las escalas de ambos grupos de estudiantes (p < 0,05). Las puntuaciones medias de las estudiantes nativas fueron más altas. Las variables “cómo limpiar la zona genital”, “material utilizado para la higiene genital”, “método de lavado de la ropa interior”, “hábitos de lavado de manos antes de ir al baño” y “menstruación regular” resultaron ser las más efectivas en términos de puntuaciones medias de la Escala de Comportamientos de Higiene Genital y sus subescalas para las estudiantes inmigrantes.

**Conclusión:**

La situación de seguridad social y la nacionalidad fueron los factores sociodemográficos más influyentes en los comportamientos de higiene genital. Sería beneficioso crear conciencia y promover cambios de comportamiento en relación con la higiene genital a través de seminarios, conferencias, talleres y eventos similares dirigidos a todas las estudiantes universitarias.

## INTRODUCCIÓN

Como fenómeno social, la migración es un tema complejo. En general, la migración se refiere al movimiento de individuos de un lugar de residencia a otro por diversas razones. Factores como la guerra y el cambio climático se encuentran entre los principales impulsores de la migración^(1)^. Desde 2011, como consecuencia de la guerra civil y el conflicto en curso en Siria, cientos de miles de ciudadanos de este país se han visto obligados a emigrar a Turquía. Según la Dirección General de Gestión de la Migración, dependiente del Ministerio del Interior de la República de Turquía, en junio de 2025, el número de sirios bajo protección temporal en Turquía era de 2,699,787, de los cuales 45,273 residían en la provincia de Mardin^(2)^.

La migración afecta a los migrantes en muchos ámbitos, uno de los cuales es la salud. Las necesidades sanitarias de los emigrantes varían en función de las condiciones de vida que tienen en sus países de origen y las que encuentran en los países a los que emigran^(1)^. En sus nuevos entornos, a menudo encuentran barreras para acceder a los servicios sanitarios. Las dificultades lingüísticas, la falta de seguro de salud y un conocimiento insuficiente de los sistemas sanitarios son algunos de los principales obstáculos para obtener servicios sanitarios sostenibles^(3)^.

Recientemente, la migración se ha examinado cada vez más en relación con el género, especialmente en estudios centrados en las mujeres. Dadas las repercusiones negativas de la migración en la salud de las mujeres, éstas pueden considerarse especialmente vulnerables^(1,4)^. Las mujeres migrantes se encuentran en una situación de riesgo en cuanto a sus necesidades de salud sexual y reproductiva. El género, como determinante sociocultural, es un factor clave de este riesgo. La desigualdad de género, uno de los determinantes sociales de la salud, afecta de diversas maneras al estado y las necesidades de salud, a las formas de acceder a los servicios sanitarios y a la prestación de dichos servicios. La desigualdad de género es especialmente evidente en el ámbito de la salud y se hace más patente en los servicios de salud reproductiva^(5)^.

El matrimonio precoz, los embarazos en la adolescencia y a edades avanzadas, la fertilidad excesiva, la falta de acceso a servicios de planificación familiar y las infecciones por falta de higiene genital son algunos de los problemas más comunes a los que se enfrentan las mujeres inmigrantes^(6)^. Un estudio que evaluaba los problemas de salud reproductiva entre las refugiadas sirias reveló que el 60% de las mujeres presentaban quejas de flujo patológico^(7)^. Según el Informe sobre Migración y Salud 2019 del Ministerio de Sanidad de la República de Turquía, existe un mayor riesgo de infecciones genitales y del tracto urinario entre los refugiados y los solicitantes de asilo^(8)^. El UNFPA Turquía lleva a cabo actividades dirigidas a empoderar a mujeres y niñas y a distribuir kits de higiene y de salud materna y reproductiva entre los refugiados^(9)^. Este estudio se elaboró partiendo de la base de que evaluar la eficacia de las intervenciones relacionadas con la higiene (por ejemplo, educación, kits de higiene) para migrantes es esencial para hacer hincapié en la sostenibilidad de los esfuerzos de ayuda. Existen estudios en Turquía y en todo el mundo sobre la determinación de los comportamientos de higiene genital de las mujeres, la investigación de los factores que influyen en los comportamientos de higiene genital y la evaluación de la relación entre los comportamientos de higiene genital y las infecciones genitales^(10-18)^. Aunque las estudiantes universitarias no representan a todas las mujeres inmigrantes, se considera que este grupo está más favorecido en términos de acceso a los servicios sanitarios y nivel educativo. Por lo tanto, se espera que los resultados aporten información sobre un segmento sociocultural específico. El objetivo de este estudio era comparar los comportamientos de higiene genital de las estudiantes nacidas en Turquía y migrantes sirias de la Universidad de Mardin Artuklu y identificar los factores culturales y sociodemográficos (como la edad, el nivel de ingresos y el estado civil) que influyen en estos comportamientos.

## MÉTODO

### Tipo de Investigación

Este estudio utilizó un diseño de investigación transversal para comparar los comportamientos de higiene genital de las estudiantes nativas turcas y las inmigrantes sirias que estudian en la Universidad Mardin Artuklu y para determinar los factores culturales y sociodemográficos (edad, nivel de ingresos, estado civil, etc.) que influyen en estos comportamientos. Se eligió el diseño transversal porque permite la evaluación práctica de las conductas de salud en una muestra más amplia, en particular cuando los estudios de seguimiento longitudinal se ven limitados por problemas como el acceso, la movilidad, las limitaciones de tiempo y las dificultades para obtener el consentimiento informado, problemas comunes cuando se investiga a poblaciones migrantes. En la investigación en salud pública, a menudo se prefieren los estudios transversales para examinar las asociaciones entre variables demográficas y conductas de salud. Son especialmente valiosos para identificar las condiciones actuales y el papel de los determinantes sociales en moldear los resultados relacionados con la salud.

### Participantes y Muestreo

La población del estudio estaba formada por mujeres estudiantes (n = 5970) matriculadas en departamentos no sanitarios (ya que se suponía que las estudiantes de los departamentos sanitarios habían recibido formación sobre higiene genital) en la Universidad de Mardin Artuklu entre 2022 y 2023. De ellos, 636 eran estudiantes sirias. El tamaño de la muestra del estudio se determinó mediante un análisis de potencia y el programa informático G*Power. El tamaño total de la muestra de 330 individuos se distribuyó equitativamente entre ambos grupos, con un nivel de significación de p = 0,05, un tamaño del efecto medio, una potencia de estudio del 99% y un valor 1-Beta fijado en 0,99 sobre la base de la d de Cohen = 0,5^(19)^. Teniendo en cuenta las limitaciones de tiempo y recursos, se eligió el método de muestreo de conveniencia para llegar al público objetivo de forma rápida y eficaz. Aunque esta elección garantizó la viabilidad del estudio, también limitó la generalizabilidad de los resultados, ya que los datos se centraron en un grupo específico de estudiantes de la Universidad Mardin Artuklu.

### Consideraciones Éticas

Se obtuvieron todos los permisos necesarios del Comité de Ética No Invasiva del Rectorado de la Universidad Mardin Artuklu (fecha: 9 de noviembre de 2022, nº: 2022/13-24) y de Karahan, quien desarrolló la Escala de Conductas de Higiene Genital (Genital Hygiene Behaviors Scale - GHBS) y evaluó su validez y fiabilidad. El estudio se llevó a cabo de conformidad con los principios recogidos en la Declaración de Helsinkİ Antes de la recogida de datos, se proporcionó a los participantes la información necesaria sobre la investigación y se obtuvo su consentimiento informado por escrito.

### Instrumentos de Recogida de Datos

El instrumento de recogida de datos constaba de dos secciones. En la primera sección, se utilizó el Cuestionario de Características Sociodemográficas (Sociodemographic Characteristics Questionnaire - SCQ) para determinar las características sociodemográficas de las participantes incluidas en el estudio, mientras que en la segunda sección, se utilizó el GHBS para evaluar las conductas de higiene genital.

– SCQ: El cuestionario fue desarrollado por los investigadores específicamente para este estudio. El SCQ consta de un total de 35 preguntas relacionadas con la edad, la nacionalidad, el lugar de residencia, el estatus de ciudadanía en caso de ser extranjero, el estado civil, si tienen hijos, el tipo de familia, el estatus de seguridad social, el nivel educativo de los padres, el estatus laboral del estudiante, el estatus de ingresos, la región donde residen durante su educación, los hábitos de fumar, la presencia de enfermedades crónicas y los comportamientos de higiene genital^(13,14,17,20,21)^.
– GHBS: Esta escala tipo Likert fue desarrollada por Karahan en 2017, y se evaluó su fiabilidad y validez. La escala se ha utilizado previamente en diferentes poblaciones y es una herramienta ampliamente utilizada para evaluar los comportamientos de higiene genital de las mujeres. Consta de 23 afirmaciones valoradas en una escala que va de “Totalmente de acuerdo” a “Totalmente en desacuerdo”, con un rango de 5 a 1. La escala comprende tres subescalas: “Hábitos de higiene general (General Hygiene Habits – GHH)” (12 primeras afirmaciones), “Hábitos de higiene menstrual (Menstrual Hygiene Habits- MHH)” (13-20 afirmaciones) y “Conciencia de hallazgos anormales (Awareness of Abnormal Findings – AAF)” (21–23 afirmaciones). En la evaluación, las afirmaciones 7, 14, 19, 20 y 23 se codifican de forma inversa, lo que permite un rango de puntuación de 23 (la más baja) a 115 (la más alta), en el que las puntuaciones más altas indican mejores conductas de higiene genital en las mujeres. El valor alfa de Cronbach de la escala original fue de 0,80^(20)^. En nuestro estudio, el valor alfa de Cronbach para la escala fue de 0,83.

### Método de Recolección de Datos

Los datos del estudio se recogieron entre marzo y mayo de 2023 mediante entrevistas personales. Los participantes que cumplían los criterios de inclusión fueron informados sobre el estudio y se obtuvo su consentimiento escrito y verbal. El llenado de los formularios de recogida de datos llevó aproximadamente entre 10 y 15 minutos.

### Criterios de Inclusión

– Mujeres estudiantes de 18 años o más
– Aceptar voluntariamente participar en la investigación
– Estudiar en departamentos distintos de los de salud
– No tener problemas de idioma turco (para las estudiantes sirias)

### Análisis de Datos

Después de que el investigador codificara los datos de este estudio, se transfirieron al software SPSS versión 16,0 para realizar los análisis necesarios.

El análisis estadístico del estudio incluyó el uso de medias, desviaciones estándar, valores mínimos y máximos, y números percentiles. Para determinar si los datos estaban distribuidos normalmente, se utilizó la prueba de Kolmogorov-Smirnov. Las puntuaciones con distribución normal se evaluaron mediante pruebas chi-cuadrado, pruebas t (pruebas t de muestras independientes) y ANOVA unidireccional. Se aplicó la prueba de Tukey, una prueba post hoc, para determinar qué grupos eran diferentes entre sí mediante ANOVA unidireccional. Los datos se analizaron mediante un modelo lineal general (MLG) para controlar posibles variables de confusión.

### RESULTADOS

La edad media de las estudiantes que participaron en el estudio fue de 23,02 ± 4,42 años (Mín: 17, Máx: 50). La edad media de las estudiantes nativas turcas fue de 23,12 ± 4,27 años (Mín: 17, Máx: 48), mientras que la edad media de las estudiantes inmigrantes fue de 23,06 ± 4,72 años (Mín: 18, Máx: 50).

Las características sociodemográficas de los participantes se presentan en la Tabla 1.

**Tabla 1:** Características sociodemográficas de los participantes Características sociodemográficas de los participantes – Mardin, Türkiye, 2023.

Características		Estudiantes nativos		Estudiantes inmigrantes		X^2^	p-valor
		n	%	n	%		
Estado civil	Casado	16	9,7	32	19,4	6,24	**< 0,05**
	Único	149	90,3	133	80,6		
Con hijos	Sí	11	6,7	26	15,8	6,84	**< 0,05**
	No	154	93,3	139	84,2		
Nivel de estudios de la madre	Analfabetos	42	25,5	29	17,6	15,33	**< 0,05**
	Graduado en educación primaria	58	35,2	36	21,8		
	Graduado en secundaria	25	15,2	37	22,4		
	Bachillerato	24	14,5	34	20,6		
	Licenciatura o más	16	9,7	29	17,6		
Nivel de estudios del padre	Analfabetos	7	4,2	12	7,3	19,79	**< 0,05**
	Graduado en educación primaria	47	28,5	18	10,9		
	Graduado en secundaria	29	17,6	48	29,1		
	Bachillerato	43	26,1	40	24,2		
	Licenciatura o más	39	23,6	47	28,5		
Zona de residencia durante los estudios	Dormitorio público	89	53,9	48	29,1	29,97	**< 0,05**
	Dormitorio privado	7	4,2	20	12,1		
	Casa de estudiantes	15	9,1	40	24,2		
	En familia	54	32,7	57	34,5		
Seguridad social	Tiene seguridad social	105	63,6	59	35,8	25,64	**< 0,05**
	No tiene seguridad social	60	36,4	106	64,2		
Total		165	100,0	165	100,0		

X^2^: prueba chi-cuadrado.

Existen diferencias estadísticamente significativas entre las estudiantes nativas turcas y las inmigrantes en cuanto al estado civil, tener hijos, el nivel educativo de los padres, la zona de residencia durante los estudios y las características de la cobertura de la seguridad social (p < 0,05).

En comparación con las estudiantes nacidas en Turquía, las estudiantes inmigrantes tienen más probabilidades de estar casadas y tener hijos (19,4%). Además, los niveles educativos de los padres de las estudiantes inmigrantes son generalmente superiores a los de los padres de las estudiantes nativas turcas. Durante el curso académico, el porcentaje de estudiantes inmigrantes que residen en residencias públicas (29,1%) es inferior al de las estudiantes nativas turcas, mientras que los porcentajes que residen en residencias privadas (12,1%) y hogares de estudiantes (24,2%) es superior. El porcentaje de estudiantes inmigrantes sin cobertura de la seguridad social es superior al de las estudiantes nativas de Turquía (64,2%).

Los comportamientos de las estudiantes en relación con la higiene genital se presentan en la Tabla 2.

**Tabla 2:** Comportamientos de las estudiantes (N = 330) en relación con la higiene genital – Mardin, Türkiye, 2023.

Características		Estudiantes nativas		Estudiantes inmigrantes		X^2^	p
		n	%	n	%		
Recibir educación sobre higiene genital	Sí	59	35,8	82	49,7	6,80	**< 0,05**
	No	77	46,7	63	38,2		
	Sí, pero insuficiente	29	17,6	20	12,1		
	Aleatoriamente	20	12,1	30	18,2		
Padece infección genital o urinaria	Sí	67	40,6	45	27,3	6,54	**< 0,05**
	No	98	59,4	120	72,7		
Menstruación regular	Sí	144	87,3	127	77,0	5,96	**< 0,05**
	No	21	12,7	38	23,0		
Uso de cualquier sustancia, crema, perfume, etc., en la zona genital	Sí	12	7,3	26	15,8	5,82	**< 0,05**
	No	153	92,7	139	84,2		
Método de depilación genital	Cera	31	18,8	46	27,9	13,23	**< 0,05**
	Hoja de afeitar	69	41,8	79	47,9		
	Depiladora	46	27,3	27	16,4		
	Cortadora	2	1,2	5	3,0		
	Sin pelo	18	10,9	8	4,8		
Presencia de secreciones malolientes	Sí	41	24,8	16	9,7	13,25	**< 0,05**
	No	124	75,2	149	90,3		
Formación sobre altas anormales	Sí	21	12,7	50	30,3	15,9	**< 0,05**
	No	144	87,3	115	69,7		
Método de lavado de la ropa interior	A alta temperatura en la máquina	127	77,0	132	80,0	6,45	**< 0,05**
	A baja temperatura en la máquina	31	18,8	18	10,9		
	A mano/por ebullición	7	4,2	15	9,1		
Total		165	100,0	165	100,0		

X^2^: prueba chi-cuadrado.

Se detectaron diferencias significativas en los hábitos de higiene y salud entre los alumnos nativos y los inmigrantes. Los alumnos inmigrantes presentaban tasas más elevadas de educación en higiene genital (49,7%). Las tasas de infecciones genitales o del tracto urinario (40,6%) y de menstruación regular (87,3%) eran más elevadas entre las estudiantes nativas turcas. Las tasas de uso de cualquier sustancia en la zona genital (15,8%) y de uso de hojilla de afeitar para depilarse la zona genital (47,9%) eran más elevadas entre las estudiantes inmigrantes. La tasa de flujo maloliente era mayor en los alumnos nativos (24,8%), y la tasa de recibir educación sobre el flujo anormal era mayor en los alumnos inmigrantes (30,3%). En cuanto al método de lavado de la ropa interior, los alumnos inmigrantes utilizan el lavado a máquina a alta temperatura (80,0%).

Las distribuciones estadísticas descriptivas de las puntuaciones de la GHBS y sus subescalas se presentan en la Tabla 3.

**Tabla 3:** Distribución estadística descriptiva de las puntuaciones de la GHBS y sus subescalas distribución estadística descriptiva de las puntuaciones de la GHBS y sus subescalas – Mardin, Türkiye, 2023.

Escala y subescalas	Estudiantes nativos					Alumnos inmigrantes					*p valor
	N	Mín.	Máx.	Media	Sd	N	Mín.	Máx.	Media	Sd	
GHBS	165	51,00	114,00	90,81	11,96	165	44,00	113,00	86,50	14,01	**< 0,05**
GHH	165	24,00	60,00	46,63	6,29	165	17,00	60,00	45,24	8,21	> 0,05
MHH	165	17,00	40,00	33,48	4,89	165	14,00	40,00	30,07	5,28	**< 0,05**
AAF	165	3,00	15,00	10,78	3,45	165	5,00	15,00	11,18	2,53	> 0,05

GHH: hábito de higiene general, MHH: hábito de higiene menstrual, AAF: conciencia de hallazgos anormales.

*Prueba t de muestras independientes.

Si bien hubo una diferencia estadísticamente significativa entre los dos grupos en las puntuaciones de las subescalas GHBS y MHH (p < 0,05), no hubo diferencia estadísticamente significativa entre los dos grupos en las puntuaciones de las subescalas GHH y AAF (p > 0,05).

Los datos se analizaron mediante un modelo lineal general (MLG) para controlar las posibles variables de confusión y se presentan en la Tabla 4.

**Tabla 4:** Efectos de las características sociodemográficas efectos de las características sociodemográficas como factores de confusión en las conductas de higiene genital – Mardin, Türkiye, 2023.

Fuente	Suma de cuadrados	df	Cuadrado medio	F	Sig.	Eta cuadrado parcial
Modelo corregido	4439,553	7	634,222	3,903	**< 0,05**	0,078
Intercepción	452629,631	1	452629,631	280,807	**< 0,05**	0,466
Estado civil	8,406	1	8,406	0,052	> 0,05	0,000
¿Tiene hijos?	9,253	1	9,253	0,057	> 0,05	0,000
Nivel educativo de la madre	383,658	1	383,658	2,361	> 0,05	0,007
Nivel de estudios del padre	98,255	1	98,255	0,605	> 0,05	0,002
¿Dónde se aloja durante el periodo educativo?	86,189	1	86,189	0,531	> 0,05	0,002
¿Tiene seguridad social?	1414,934	1	1414,934	8,708	**< 0,05**	0,026
Nacionalidad	1144,861	1	1144,861	7,046	**< 0,05**	0,021
Error	52233,344	322	162,495			
Total	56762,897	329				

Se observó que la seguridad social y la nacionalidad tenían un efecto confundente significativo en las conductas de higiene genital (p < 0,05). Los estudiantes con seguridad social y los nacidos en el país tenían puntuaciones significativamente más altas en las conductas de higiene genital. El eta cuadrado parcial (η^2^) muestra que los efectos de confusión de las variables seguridad social (2,6%) y nacionalidad (2,1%) son pequeños (η^2^ < 0,06). Otras variables (estado civil, tener hijos, nivel de educación de los padres y lugar de residencia durante el período de educación) no confundieron significativamente las conductas de higiene genital (p > 0,05).

La Tabla 5 presenta la comparación de las puntuaciones totales de la escala y de las subescalas entre alumnos nativos e inmigrantes en función de determinadas características.

**Tabla 5:** Comparación de las puntuaciones totales de la escala y las subescalas entre alumnos nativos e inmigrantes en función de determinadas características – Mardin, Türkiye, 2023.

Características		Alumnos nativos				Alumnos inmigrantes			
		GHBS	GHH	MHH	AAF	GHBS	GHH	MHH	AAF
Edad	17–21 Edad^a^	89,23 ± 12,99	45,25 ± 6,76	33,50 ± 5,24	10,47 ± 3,63	86,09 ± 13,40	45,02 ± 8,04	30,36 ± 5,04	10,70 ± 2,49
	22–26 Edad^b^	90,45 ± 11,16	46,80 ± 5,93	33,21 ± 4,85	10,43 ± 3,38	85,55 ± 13,65	44,86 ± 7,98	29,48 ± 5,34	11,20 ± 2,30
	27–31 Edad^c^	102,40 ± 7,64	52,30 ± 5,16	36,70 ± 3,43	13,40 ± 2,11	92,45 ± 13,95	48,09 ± 9,19	31,90 ± 3,59	12,45 ± 2,94
	32 Edad^d^↑	92,25 ± 9,32	46,91 ± 5,23	32,66 ± 3,84	12,66 ± 2,60	88,91 ± 19,36	46,16 ± 10,0	29,91 ± 7,51	12,83 ± 2,82
	Análisis estadístico	F = 3,79 **p = 0,01**	F = 3,79 **p = 0,01**	F = 1,66 p = 0,17	F = 3,73 **p = 0,01**	F = 0,90 p = 0,40	F = 0,55 p = 0,64	F = 0,790 p = 0,49	F = 3,67 **p = 0,01**
	Diferencia	a–c, b–c, c–a–b	a–b–c, b–a–c, c–a–b		b–c, c–b				a–d
Estado de ingresos*	Ingresos inferiores a los gastos^a^	87,17 ± 12,31	45,40 ± 6,36	32,56 ± 4,78	9,21 ± 3,56	85,43 ± 14,61	44,45 ± 8,73	29,76 ± 5,33	11,08 ± 2,66
	Los ingresos equivalen a los gastos^b^	91,90 ± 11,88	46,94 ± 6,37	33,64 ± 5,00	11,32 ± 3,08	87,81 ± 13,50	46,20 ± 7,74	30,38 ± 5,13	11,22 ± 2,43
	Ingresos superiores a los gastos^c^	96,25 ± 10,39	48,45 ± 5,46	35,12 ± 4,41	12,66 ± 2,94	83,46 ± 14,28	42,61 ± 8,17	29,46 ± 6,29	11,38 ± 2,69
	Análisis estadístico	F = 5,91 p = **0,003**	F = 2,22 p = 0,111	F = 2,45 p = 0,089	F = 11,91 p = **0,000**	F = 0,87 p = 0,421	F = 1,48 p = 0,229	F = 0,33 p = 0,719	F = 0,09 p = 0,908
	Diferencia	a–b–c, b–a, c–a			a–b–c, b–a, c–a				
Estado civil**	Casado	93,50 ± 11,87	48,18 ± 6,02	32,62 ± 5,76	12,68 ± 2,57	87,59 ± 16,60	45,28 ± 9,86	30,09 ± 5,58	12,21 ± 2,81
	Individual	90,62 ± 11,83	46,46 ± 6,32	33,57 ± 4,80	10,58 ± 3,47	86,24 ± 13,37	45,24 ± 7,81	30,06 ± 5,23	10,93 ± 2,40
	Análisis estadístico	t = 0,92 p = 0,357	t = 1,04 p = 0,299	t = 0.74 p = 0,461	t = 2,35 **p = 0,020**	t = 0,49 p = 0,625	t = 0,25 p = 0,980	t = 0,25 p = 0,980	t = 2,38 **p = 0,022**
Tipo de familia**	Extendida	87,76 ± 11,42	45,61 ± 6,16	32,46 ± 4,51	9,68 ± 3,84	83,94 ± 15,76	43,40 ± 9,50	29,69 ± 5,92	10,84 ± 2,67
	Nuclear (madre, padre e hijos)	92,15 ± 11,80	47,03 ± 6,33	33,88 ± 4,99	11,22 ± 3,19	87,92 ± 12,80	46,27 ± 7,25	30,28 ± 4,91	11,36 ± 2,45
	Análisis estadístico	t = –2,17 **p = 0,031**	t = –1,31 p = 0,193	t = –1,69 p = 0,092	t = –2,65 **p = 0,009**	t = 1,66 p = 0,101	t = –2,01 **p = 0,047**	t = –0,65 p = 0,518	t = –1,27 p = 0,207
Seguridad social**	Tiene seguridad social	92,58 ± 10,63	47,48 ± 5,71	33,92 ± 4,19	11,17 ± 3,49	89,84 ± 12,17	47,00 ± 7,11	31,11 ± 4,95	11,72 ± 2,63
	No tiene seguridad social	87,96 ± 13,25	45,13 ± 7,00	32,71 ± 5,88	10,11 ± 3,29	84,64 ± 14,66	44,27 ± 8,64	29,49 ± 5,40	10,87 ± 2,43
	Análisis estadístico	t = 2,30 **p = 0,023**	t = 2,33 **p = 0,021**	t = 1,40 p = 0,165	t = 1,90 p = 0,059	t = 2,44 **p = 0,016**	t = 2,18 **p = 0,031**	t = 1,91 p = 0,058	t = 2,09 **p = 0,038**
Uso de cualquier sustancia, crema, perfume, etc., en la zona genital	Sí	96,66 ± 8,53	50,58 ± 3,60	34,08 ± 5,20	12,00 ± 2,95	82,04 ± 19,03	42,00 ± 11,63	29,35 ± 6,55	10,69 ± 2,91
	No	90,45 ± 11,95	46,32 ± 6,37	33,44 ± 4,88	10,69 ± 3,48	87,34 ± 12,78	45,86 ± 7,30	30,21 ± 5,04	11,27 ± 2,46
	Análisis estadístico	t = 1,76 p = 0,08	t = 3,67 **p = 0,00**	t = 0,44 p = 0,66	t = 1,27 p = 0,21	t = –1,36 p = 0,18	t = –1,63 p = 0,11	t = –0,64 p = 0,53	t = –1,07 p = 0,29
El método de depilación genital*	Cera^a^	89,77 ± 14,60	45,54 ± 7,29	33,38 ± 5,10	10,83 ± 3,64	88,95 ± 15,05	46,50 ± 8,44	30,73 ± 5,68	11,71 ± 2,44
	Hoja de afeitar^b^	87,95 ± 11,37	45,26 ± 6,23	32,50 ± 4,83	10,18 ± 3,45	85,67 ± 13,20	44,94 ± 7,77	29,92 ± 4,96	10,79 ± 2,52
	Depiladora^c^	94,04 ± 9,09	48,28 ± 5,70	34,51 ± 4,09	11,24 ± 3,41	87,81 ± 12,29	46,37 ± 7,63	30,11 ± 4,89	11,33 ± 2,61
	Clipper^d^	82,50 ± 20,50	43,00 ± 2,82	28,50 ± 12,08	11,00 ± 5,65	69,40 ± 13,52	35,40 ± 10,73	24,20 ± 2,94	9,80 ± 2,48
	Sin pelo	97,22 ± 10,17	50,00 ± 4,22	35,38 ± 5,08	11,83 ± 2,93	86,87 ± 16,50	43,37 ± 8,81	31,25 ± 7,06	12,25 ± 2,37
	Análisis estadístico	F = 3,70 **p = 0,007**	F = 3,48 **p = 0,009**	F = 2,47 **p = 0,046**	F = 1,14 p = 0,341	F = 2,42 **p = 0,050**	F = 2,40 **p = 0,050**	F = 1,88 p = 0,117	F = 1,75 p = 0,142
	Diferencia	b–e	b–e	e–b		a–d	a–d, c–d		
Método de lavado de la ropa interior*	A alta temperatura en la máquina^a^	91,51 ± 11,14	46,93 ± 6,05	33,54 ± 4,77	11,03 ± 3,26	88,13 ± 13,84	46,03 ± 8,24	30,68 ± 5,02	11,41 ± 2,44
	A baja temperatura en la máquina^b^	86,87 ± 13,12	44,51 ± 6,66	32,61 ± 5,27	9,74 ± 4,00	78,11 ± 11,44	41,72 ± 6,71	26,77 ± 5,66	9,61 ± 2,72
	A mano/por ebullición^c^	97,71 ± 11,25	50,42 ± 7,13	36,28 ± 4,71	11,00 ± 3,26	82,20 ± 14,59	42,60 ± 8,39	28,60 ± 5,69	11,00 ± 2,56
	Análisis estadístico	F = 3,21 **p = 0,043**	F = 3,26 **p = 0,041**	F = 1,66 p = 0,193	F = 1,77 p = 0,174	F = 5,07 **p = 0,007**	F = 3,11 **p = 0,047**	F = 5,23 **p = 0,006**	F = 4,22 p = 0,016
	Diferencia	a–b	b–a			a–b	a–b	a–b	
Menstruación regular**	Sí	91,40 ± 11,77	46,85 ± 6,29	33,72 ± 4,77	10,81 ± 3,42	88,53 ± 13,22	46,41 ± 7,80	30,77 ± 5,02	11,33 ± 2,50
	No	87,47 ± 11,91	45,09 ± 6,24	31,80 ± 5,44	10,57 ± 3,69	79,71 ± 14,61	41,34 ± 8,44	27,71 ± 5,54	10,65 ± 2,60
	Análisis estadístico	t = 1,425 p = 0,156	t = 1,197 p = 0,233	t = 1,690 p = 0,093	t = 0,307 p = 0,759	t = 3,522 **p = 0,001**	t = 3,450 **p = 0,001**	t = 3,226 **p = 0,002**	t = 1,457 p = 0,680
Presencia de vertidos fétidos**	Sí	85,80 ± 11,57	45,90 ± 6,64	31,87 ± 4,73	8,02 ± 3,51	78,93 ± 13,01	41,93 ± 7,66	27,31 ± 5,91	9,68 ± 2,98
	No	92,58 ± 11,46	46,87 ± 6,19	34,01 ± 4,84	11,70 ± 2,91	87,31 ± 13,91	45,60 ± 8,21	30,36 ± 5,15	11,34 ± 2,44
	Análisis estadístico	t = 3,28 **p = 0,001**	t = –0,85 p = 0,395	t = 2,46 **p = 0,015**	t = 6,65 **p = 0,000**	t = 2,30 **p = 0,023**	t = 1,71 p = 0,090	t = 2,22 **p = 0,028**	t = 2,52 **p = 0,013**

*ANOVA de una vía y test de Tukey (post hoc).

**Test t de muestras independientes.

En el presente estudio, las conductas de higiene genital y sus subescalas no se vieron influidas por variables como el nivel de educación del padre, el nivel de educación de la madre, tener hijos, la zona de residencia durante la educación, la situación laboral, recibir educación sobre higiene genital, el tipo de retrete utilizado, el dolor durante la menstruación, el uso de cualquier sustancia, crema, perfume, etc., en la zona genital, o la formación sobre flujo anormal (p > 0,05).

## DISCUSIÓN

En nuestros resultados, las diferencias estadísticamente más significativas en las conductas y subescalas de higiene genital, tanto en individuos nativos como inmigrantes, se encontraron en cuanto al método de limpieza de la zona genital, el método de lavado de la ropa interior, la seguridad social y la presencia de flujo maloliente. El hecho de que se hayan llevado a cabo trabajos y proyectos de ayuda para inmigrantes en Turquía puede tener un efecto positivo en algunos resultados^(22)^.

Se observó una diferencia entre el nivel de ingresos de las estudiantes nativas turcas y las puntuaciones medias de la GHBS y las puntuaciones de la subescala de conocimiento de hallazgos anormales. Estudios realizados en lugares diferentes^(14,21)^ han demostrado que, a medida que aumentan los ingresos, también aumentan las puntuaciones totales de la escala de comportamiento de higiene genital. El hecho de que a las personas con mayores ingresos les resulte más fácil acceder a productos higiénicos puede haber influido positivamente en sus conductas de higiene genital.

Aunque había diferencias significativas entre las puntuaciones de la subescala de tipo de familia, hábitos generales de higiene y conocimiento de hallazgos anormales de las estudiaantes nacidas en Turquí, no se observaron tales diferencias en las puntuaciones de la subescala de hábitos generales de higiene de los inmigrantes. Las puntuaciones medias totales de los que vivían en familias nucleares eran más altas. La familia nuclear, definida como una unidad familiar formada únicamente por los padres y sus hijos, puede tener un impacto positivo en los comportamientos relacionados con la higiene. Esta estructura facilita una mayor privacidad de las personas, mejora el acceso a los productos de higiene y reduce el número de personas que comparten espacios comunes como baños y retretes, promoviendo así la mejora de las prácticas higiénicas. En un estudio realizado con niñas adolescentes escolarizadas en Nepal, el 67% de las participantes tenían una familia nuclear. Entre las que tenían de uno a cuatro miembros en la familia, el porcentaje de individuos que mostraban sólidas prácticas de higiene menstrual era mayor que entre las que tenían cinco o más miembros^(19)^. En un estudio en el que se examinaron las conductas de higiene genital de las mujeres que acudían al Centro de Salud Comunitario de Mardin, las mujeres con familia nuclear obtuvieron puntuaciones medias más altas en las conductas de higiene genital^(18)^. Los resultados de nuestro estudio coinciden en su mayor parte con los de la bibliografía.

En nuestro estudio, las puntuaciones medias de la escala de conductas de higiene genital y sus subescalas fueron generalmente más altas en las que tenían seguridad social. En diferentes estudios realizados en mujeres desfavorecidas y no desfavorecidas, se observó que las puntuaciones de las conductas de higiene genital de las que tenían seguridad social eran más altas, como en nuestro estudio^(12,18,22)^. El menor coste de la asistencia sanitaria para las personas con seguridad social las orienta hacia las instituciones sanitarias y les permite acceder a la información necesaria por parte de los profesionales sanitarios.

En el presente estudio, el 41,8% de las estudiantes nativas turcas y el 47,9% de las estudiantes inmigrantes declararon limpiarse las zonas genitales con hojillas de afeitar. En un estudio en el que participaron mujeres estudiantes que vivían en una residencia universitaria estatal, el porcentaje de las que limpiaban sus zonas genitales con hojillas de afeitar (35,9%) era mayor que el de las que lo hacían con cera (34,3%)^(21)^. Factores como las posibilidades económicas de las estudiantes, la necesidad de convivir en lugares muy concurridos y la falta de conocimientos sobre higiene genital pueden haber afectado a los resultados obtenidos. Este estudio no evaluó una relación directa entre los métodos de depilación y las tasas de infección; sin embargo, la bibliografía informa de que el uso de hojillas de afeitar puede aumentar el riesgo de infección al dañar la integridad de la piel (picor, irritación, eczema localizado, pelos encarnados, cortes microscópicos, etc.)^(23,24)^. Por lo tanto, la elevada tasa de uso de hojillas de afeitar es digna de mención en términos de riesgo potencial. Dado que el estudio tiene un diseño transversal, no es posible establecer una relación causal; sin embargo, esta situación puede considerarse un tema importante para futuras investigaciones.

Las estudiantes nativas que lavan su ropa interior a mano con agua hirviendo y las estudiantes inmigrantes que la lavan a altas temperaturas en lavadoras presentan, en general, puntuaciones más altas en el GHBS y sus subescalas. Puede haber dos posibles explicaciones para este hallazgo. En primer lugar, los inmigrantes que no han adquirido la nacionalidad turca no tienen derecho a residir en residencias públicas y, por lo tanto, es posible que vivan en residencias privadas, residencias de estudiantes o con familias, lugares que pueden ofrecer mejores condiciones higiénicas que las residencias públicas. En segundo lugar, sus respuestas pueden reflejar conocimientos precisos y prácticas de higiene mejoradas adquiridos a través de componentes educativos de programas de ayuda humanitaria orientados a la salud. En un estudio de Baş et al.^(25)^, realizado en instituciones de crédito y residencias universitarias, el 54,80% de las estudiantes, y en un estudio de Kartal et al.^(17)^, realizado en estudiantes de partería, el 91,40% de las estudiantes afirmaron que lavaban su ropa interior en la lavadora. En un estudio de Toraman et al.^(26)^ en el que se examinaron los comportamientos de higiene genital de las mujeres que vivían en una casa de huéspedes para mujeres, el 20,70% de las mujeres lavaban su ropa interior a altas temperaturas en la máquina. Los estudios de la bibliografía pueden mostrar variabilidades, que pueden atribuirse a la limitación de recursos, las diferencias culturales y la falta de conocimientos sobre el tema.

La tasa de irregularidades menstruales entre las estudiantes inmigrantes es del 23%, mientras que entre las nativas de Turquía es del 12,7%. Las que experimentaban una menstruación regular tenían puntuaciones más altas en GHBS, hábitos generales de higiene e higiene menstrual total. Las irregularidades menstruales en estudiantes inmigrantes pueden estar relacionadas con el estrés psicológico, las barreras para acceder a los servicios sanitarios y los problemas de adaptación cultural, principalmente las dificultades económicas. En un estudio de Janoowalla et al.^(27)^ realizado en Ruanda, el 69,10% de las adolescentes, y en un estudio de Belayneh y Mekuriaw^(28)^ en el que se examinaron los conocimientos sobre higiene menstrual de las adolescentes del sur de Etiopía, el 40,30% declaró experimentar menstruaciones regulares. El hecho de que los dos países de los estudios de la muestra tengan niveles socioeconómicos muy bajos sugiere que las irregularidades menstruales pueden deberse a problemas derivados de dificultades económicas en particular. Nuestro estudio concuerda con la bibliografía. Sería beneficioso establecer mecanismos integrales de apoyo mediante la colaboración entre universidades, organizaciones no gubernamentales (ONG) y el Ministerio de Sanidad para resolver estos problemas.

De todas las estudiantes, el 75,20% de las nativas y el 90,30% de las inmigrantes declararon no tener flujo con olor sospechoso. La presencia de flujo maloliente suele afectar a los comportamientos de higiene genital, higiene menstrual y la conciencia de hallazgos anormales. Muzayyanatul y Wulan^(29)^ informaron una menor tasa de flujo vaginal patológico (3,50%) entre las estudiantes que mantenían la higiene genital. En un estudio sobre la prevención de las infecciones del tracto genitourinario entre las estudiantes adolescentes, se informó que el flujo vaginal representaba el 18,60% de las quejas reportadas^(30)^. Curiosamente, se ha observado que algunos comportamientos higiénicos entre las estudiantes refugiadas son más positivos de lo esperado. Teniendo en cuenta los retos socioeconómicos a los que se enfrentan los grupos de inmigrantes, esta situación puede parecer contradictoria a primera vista. Sin embargo, los proyectos de ayuda centrados en la salud, los seminarios de concientización y las actividades informativas dirigidas a los refugiados sirios que viven en Turquía pueden haber apoyado el proceso de adaptación cultural al fomentar la adopción de hábitos sanitarios locales. Por otro lado, dado que los datos utilizados en nuestro estudio son autoinformados, también debe tenerse en cuenta la posibilidad de que los participantes se inclinen a dar respuestas socialmente aceptables (sesgo de relato), especialmente en cuestiones delicadas.

### Limitaciones del Estudio

- Este estudio se limita a una muestra de estudiantes universitarias. Los niveles educativos de las mujeres nativas de Turquía y migrantes pueden diferir, lo que puede afectar a sus hábitos de higiene.
- Dado que los datos se basan en los autoinformes de las estudiantes, éstas pueden haber dado respuestas consideradas socialmente más aceptables. Esto puede dar lugar a sesgos.
- En este estudio, la duración de tiempo viviendo en Turquía y el nivel de adaptación cultural de las estudiantes inmigrantes no se incluyeron en el ámbito de la evaluación. Sin embargo, estas variables pueden afectar tanto a los comportamientos higiénicos como a los autoinformes de los participantes. Tener en cuenta estos factores en futuros estudios permitirá un análisis más exhaustivo y profundo de los resultados.

## CONCLUSIÓN

En conclusión, las diferencias en los hábitos de higiene entre alumnas nativas e inmigrantes pueden deberse a factores culturales, educativos, socioeconómicos, etc. El desarrollo y la sostenibilidad de políticas educativas y sanitarias que tengan en cuenta estas diferencias pueden desempeñar un papel importante en la mejora de los niveles de higiene de las estudiantes nativas turcas e inmigrantes.

Los estudios que comparan los hábitos de higiene de estudiantes nativas e inmigrantes siguen siendo limitados. Además, como este estudio utilizó un diseño transversal, no pudo captar los cambios de comportamiento a lo largo del tiempo. Por lo tanto, se recomiendan futuros estudios longitudinales que evalúen la eficacia de la educación en higiene y las intervenciones específicas para comprender mejor las tendencias temporales y apoyar las prácticas sanitarias basadas en la evidencia entre poblaciones estudiantiles diversas.

Los programas educativos basados en intervenciones que se desarrollen en el futuro tanto para las estudiantes nativas turcas como para las inmigrantes ofrecen un importante campo de investigación en cuanto al fortalecimiento de las conductas de higiene genital y la creación de una concienciación sostenible. En este contexto, es muy importante desarrollar estrategias de promoción de la salud dirigidas a las poblaciones migrantes. Programas educativos culturalmente sensibles, políticas públicas que garanticen el acceso a productos y servicios de higiene - especialmente para las mujeres sin seguridad social- y campañas de sensibilización dirigidas a reducir el estigma pueden mejorar las condiciones de higiene y contribuir a reducir las desigualdades en el ámbito de la salud reproductiva entre estos grupos vulnerables.

## Data Availability

Los datos que respaldan los resultados de este estudio transversal se recopilaron mediante entrevistas presenciales utilizando un cuestionario sobre características sociodemográficas y la Escala de Conductas de Higiene Genital (GHBS). Estos conjuntos de datos no están disponibles públicamente debido a la confidencialidad de los participantes y a restricciones éticas, pero pueden solicitarse al autor correspondiente previa solicitud razonable.
